# Platinum–Dysprosium Alloys as Oxygen Electrodes in Alkaline Media: An Experimental and Theoretical Study

**DOI:** 10.3390/nano12142318

**Published:** 2022-07-06

**Authors:** Jadranka Milikić, Nikola Nikolić, Diogo M. F. Santos, Daniele Macciò, Adriana Saccone, Mabkhoot Alsaiari, Mohammed Jalalah, M. Faisal, Farid A. Harraz, Yizhao Li, Abu Bakr Nassr, Igor Pašti, Biljana Šljukić

**Affiliations:** 1 Faculty of Physical Chemistry, University of Belgrade, Studentski trg 12–16, 11158 Belgrade, Serbia; jadranka@ffh.bg.ac.rs (J.M.); nikolan@kth.se (N.N.); 2 Center of Physics and Engineering of Advanced Materials, Laboratory for Physics of Materials and Emerging Technologies, Chemical Engineering Department, Instituto Superior Técnico, Universidade de Lisboa, 1049-001 Lisbon, Portugal; diogosantos@tecnico.ulisboa.pt; 3 Dipartimento di Chimica e Chimica Industriale (DCCI), Università Degli Studi di Genova, Via Dodecaneso 31, I-16146 Genova, Italy; maccio@chimica.unige.it (D.M.); adriana.saccone@unige.it (A.S.); 4 Promising Centre for Sensors and Electronic Devices (PCSED), Advanced Materials and Nano-Research Centre, Najran University, Najran 11001, Saudi Arabia; mamalsaiari@nu.edu.sa (M.A.); msjalalah@nu.edu.sa (M.J.); mfahsan@nu.edu.sa (M.F.); faharraz@nu.edu.sa (F.A.H.); 5 Empty Quarter Research Unit, Department of Chemistry, Faculty of Science and Arts at Sharurah, Najran University, Najran 61441, Saudi Arabia; 6 Department of Electrical Engineering, Faculty of Engineering, Najran University, Najran 61441, Saudi Arabia; 7 Nanomaterials and Nanotechnology Department, Central Metallurgical Research and Development Institute (CMRDI), Cairo 11421, Egypt; 8 Yangtze Delta Region Institute (Huzhou), University of Electronic Science and Technology of China, Huzhou 313001, China; yizhao@csj.uestc.edu.cn; 9 Electronic Materials Research Department, Advanced Technology and New Materials Research Institute (ATNMRI), City of Scientific Research and Technological Applications (SRTA-City), New Borg Al-Arab City 21934, Egypt; abubakramine@gmail.com

**Keywords:** oxygen reduction reaction, oxygen evolution reaction, platinum–dysprosium alloys, fuel cells, local coordination, DFT analysis

## Abstract

Platinum–dysprosium (Pt–Dy) alloys prepared by the arc melting technique are assessed as potential electrodes for the oxygen reduction reaction (ORR) using voltammetry and chronoamperometry in alkaline media. A relatively small change (10 at.%) in the alloy composition brought a notable difference in the alloys’ performance for the ORR. Pt_40_Dy_60_ electrode, i.e., the electrode with a lower amount of Pt, was identified to have a higher activity towards ORR as evidenced by lower overpotential and higher current densities under identical experimental conditions. Furthermore, DFT calculations point out the unique single-atom-like coordination and electronic structure of Pt atoms in the Pt_40_Dy_60_ surface as responsible for enhanced ORR activity compared to the alloy with a higher Pt content. Additionally, Pt–Dy alloys showed activity in the oxygen evolution reaction (OER), with the OER current density lower than that of pure Pt.

## 1. Introduction

The oxygen reduction reaction (ORR) has become one of the most studied electrochemical reactions due to its fundamental complexity and great importance in practical applications [1,2]. Specifically, different technologies, including metal-air batteries and fuel cells (FCs), are based on this reaction [3]. The mechanism of the ORR is primarily determined by the electrode material [4] and electrolytic solution [5]. Alkaline media have the advantages of improved electrode materials’ stability due to usually less corrosive electrolytes and weaker specific adsorption of anions onto the electrode surface, including platinum (Pt) ones [5,6]. Consequently, alkaline ORR is often employed as a cathode reaction in the above-mentioned electrochemical technologies, as the high pH also allows the use of thermodynamically stable transition metals and rare earth metals. However, there are drawbacks to using carbon-supported materials (e.g., Pt/C) as fuel cell cathodes in alkaline media, as the carbon support corrodes at high pH values [6] or metal nanoparticles could detach. Moreover, the ORR kinetic limitations in alkaline fuel cells (AFCs) [7] yield high cathode overpotential and lower the energy efficiency and cell power density. The sluggish ORR rate at the AFC cathode, limiting their commercialization, has spurred extensive fundamental studies of the ORR mechanism, as well as an extensive search for active and selective electrocatalysts. Pt is the most efficient electrocatalyst for ORR [8] as it is stable and promotes the direct four-electron reaction pathway in alkaline media (Equation (1)).

O_2_ + 2H_2_O + 4e^−^ → 4OH^−^   E^0^ = 0.401 V vs. SHE (1)

Still, a rather strong binding of O_2_ and other ORR intermediates on Pt surfaces leads to energetically unfavorable removal of OH_ads_, resulting in slow ORR kinetics on Pt electrodes and cell voltage loss [9,10,11]. Furthermore, widespread use of AFCs for practical applications requires a significant reduction in the amount of costly Pt. Even though progress has been made in this respect, a further reduction through the development of more active and stable electrocatalysts is crucial.

The main approaches in developing ORR catalysts for the low-temperature FC cathode involve the reduction in the Pt loading while keeping the same level of power density by alloying it, decreasing Pt particles size to nanoscale, or using structures with higher Pt utilization efficiency [12]. The use of binary electrocatalysts for ORR was first suggested in 1980 [13], with Pt-V/C showing enhanced activity and durability for the ORR compared to a pure Pt electrocatalyst. For the last few years, considerable progress has been made in understanding the ORR on Pt and Pt-based bimetallic electrocatalysts. Several investigations have been carried out to determine the role of the presence of other metals on the electrocatalytic activity [14,15,16], and much of this experimental work has indicated that the activity of Pt-based bimetallic electrocatalysts towards the ORR in alkaline media is at least as good as that of pure Pt. The presence of other elements changes the Pt-Pt bond lengths and coordination, as well as the electronic structure of the Pt 5d orbital [17,18]. Thus, by altering the electronic structure, chemical reactivity toward ORR intermediates is also changed, ultimately leading to the modulation and improvement of catalytic activity [17,19].

Most of the reported Pt-based alloys for ORR catalysis focus on 3d metals, with late 3d metals showing the best performance [20,21]. However, bimetallic alloys of Pt or palladium with rare earth (RE) metals scandium (Sc) or yttrium (Y) were suggested as novel electrocatalysts for the ORR based on density functional theory (DFT) calculations [22]. In fact, the electrochemical measurements have confirmed the improved activity of polycrystalline Pt_3_Sc and Pt_3_Y electrodes compared to pure Pt by a factor of 1.5–1.8 and 6–10, respectively, in the range 0.90–0.87 V [22]. Nevertheless, this direction in search for new ORR catalysts was not investigated in detail as much as for Pt-3d element alloys, suggesting there is a large compositional space of Pt-RE systems that can contain some exceptional ORR catalysts. The identification of Pt-Sc and Pt-Y systems as novel ORR catalysts is an excellent example of synergy between theory and experiment in the search for novel electrocatalytic systems, with many studies going in the same direction [23,24]. Other examples can be found in the literature, focusing on alkaline media, in contrast to the majority of works emphasizing acidic ORR catalysis. One example is the identification of a Pt–In system as a superior ORR catalyst in alkaline media [25]. However, the alloys of Pt and p-elements are not traditionally considered ORR catalysts but rather oxidation catalysts for small organic molecules.

Platinum-group metals (PGMs) are also the most examined electrocatalysts for oxygen evolution reactions (OER). Thus far, the most efficient OER electrocatalysts are iridium and ruthenium oxide (IrO_2_ and RuO_2_) [26]. Still, noble metal oxides have drawbacks of low OER stability and high cost [26]. Thus, PGM electrocatalysts show promise of decreased OER overpotential and improved OER activity [26,27]. Reier et al. determined the number of electrochemically available surface sites (NEASS) of bulk Pt, Ru, and Ir catalysts by CO stripping experiments where Pt and Ir gave similar values of NEASS [28].

Herein, the electrocatalytic activity for the ORR and OER of platinum–dysprosium (Pt–Dy) alloy electrodes of two different compositions has been studied in alkaline solution in the 25–65 °C temperature range by linear scan voltammetry (LSV) and chronoamperometry (CA). Thus, partial replacement of Pt by Dy while keeping the ORR activity unchanged could significantly reduce the catalyst costs [29]. Furthermore, DFT calculations were carried out, revealing single atom-like catalysis in highly active Pt–Dy alloy.

## 2. Materials and Methods

Pt–Dy alloys at 50 at.% Dy (Pt_50_Dy_50_) and 60 at.% Dy (Pt_40_Dy_60_) nominal composition, about 3 g each, were prepared by arc melting under an argon atmosphere of 0.7 bar, starting from stoichiometric amounts of Pt (99.99 wt.%, Johnson Matthey and Co. Ltd., London, UK) and Dy (99.9 wt.%, Koch Chemical Ltd., Hertford, UK). The samples were remelted up to four times to ensure homogeneity.

To check the alloys’ morphology, the existing phases, and their composition prior to the electrochemical tests, the alloys were characterized by X-ray powder diffraction (XRPD), scanning electron microscopy (SEM), and energy dispersive X-ray analysis (EDX). Details on these characterization techniques and the phase diagram investigation of the Pt–Dy system are described in the authors’ previous works [30,31].

The Pt–Dy alloy at 50 at.% Dy was found to be an almost single-phase sample (constituted by the equiatomic compound Pt–Dy, presenting a small homogeneity range) while the Pt–Dy sample at 60 at.% Dy was found to be a two-phase alloy, constituted by the two intermetallic compounds Pt_4_Dy_5_ and Pt_3_Dy_5_. Additionally, the phase diagram investigations of the alloys were performed by differential thermal analysis (DTA) [31]. The alloy at 50 at.% Dy has a high melting point (congruent melting at about 1520 °C). The Pt–Dy phase diagram presents high melting point alloys, especially in the range of 50 to 100 at.% Pt. In the range richer in Dy, the temperature of the alloys decreased to 1020 °C (Dy-rich eutectic) (Pt_40_Dy_60_ melts at about 1430 °C).

The prepared Pt–Dy alloy buttons were silver glued to copper (Cu) cables, embedded in epoxy resin and polished until reaching well-defined flat surface discs. Before the experiments, the electrodes were further polished to a mirror finish.

All electrochemical measurements were carried out on PAR 273A potentiostat (Princeton Applied Research, Inc., Oak Ridge, USA) controlled by PowerSuite software in a one-compartment glass cell of 100 mL volume of 1 M sodium hydroxide (NaOH, 99%, AnalaR) as a supporting electrolyte. Pt–Dy alloy electrode (geometric area of ca. 0.25 cm^2^) was employed as a working electrode, with Pt foil and saturated calomel electrode (SCE, Radiometer Analytical) serving as a counter and a reference electrode, respectively. All potentials in this paper are presented versus the reversible hydrogen electrode (RHE).

The electrochemical characterization was conducted by running cyclic voltammetry (CV) in N_2_-saturated solutions. For the ORR studies, O_2_ (purity 99.995%, Messer) was bubbled into the solution for 10 min before each measurement while keeping a gentle O_2_-saturated atmosphere during the measurement itself as well. Linear scan voltammetry (LSV) studies were performed using polarization rates in the 0.01–1 V s^–1^ range. The temperature influence on the ORR at Pt_40_Dy_60_ electrode was investigated by controlling the cell temperature (25–65 °C) with water circulation using a Haake F3 thermostatic bath. In addition, LSV was used for oxygen evolution reaction (OER) studies at a scan rate of 20 mV s^−1^ and 25 °C.

CA experiments were conducted in O_2_-saturated 1 M NaOH solution at a potential near the O_2_ reduction peak potential for the corresponding electrode for 1 h. Furthermore, the selectivity towards the ORR was examined by recording CA curves for 180 s, with 1 mL of fuel solution (0.03 M NaBH_4_) being added after 60 s. LSVs were also recorded in the presence of NaBH_4_.

For computational details, VASP [32,33] was employed to perform all the density functional theory (DFT) calculations within the generalized gradient approximation (GGA) using the Perdew–Burke–Ernzerhof (PBE) [34] formulation. We have chosen the projected augmented wave (PAW) potentials [35] to describe the ionic cores. The kinetic energy cutoff of 450 eV for the plane-wave basis was used. Partial occupancies of the Kohn−Sham orbitals were allowed using the Gaussian smearing method and a width of 0.05 eV. The electronic energy was considered self-consistent when the energy change was smaller than 10^−5^ eV. Geometry optimization was considered convergent when the energy change was smaller than 0.03 eV/Å. The Brillouin zone was sampled with 3 × 3 × 1 Monkhorst mesh [36]. Five-layer slabs of both studied alloys were studied with 20 Å thickness between the slabs to avoid the interactions of periodic images.

Gibbs free energy can be obtained by adding corrections including entropic (*TS*) and zero-point energy (ZPE) to calculate DFT energy so that Δ*G* = Δ*E*_DFT_ + ΔZPE *− T*Δ*S* − e*U*, where *E*_DFT_ is the calculated DFT reaction energy, ΔZPE is the change in ZPE calculated from the vibrational frequencies, and Δ*S* is the change in the entropy referring to thermodynamics databases. The electrode potential is adopted with respect to the RHE, which makes the standard electrochemical potential of the electron involved in the reaction (*G*_e_) equal to −e*U*, and the standard electrochemical potential of the proton (*G*_H^+^_) equal to that of the hydrogen atom in gaseous H_2_ (1/2*G*_H_2__). Considering that the triplet state of the O_2_ molecule is poorly described in the current DFT scheme, the free energy of the O_2_ molecule was derived according to *G*_O_2__ = 2*G*_H_2_O_ − 2*G*_H_2__ + 4.92 (in eV), as suggested by Nørskov et al. [37]. Although the problem of O_2_ description can be overcome by using higher-level theories and the cluster approach [38,39], here we have stuck to periodic DFT-PBE calculation, as usually performed for extended surfaces.

## 3. Results

### 3.1. ORR Experimental Study

**Figure 1:**
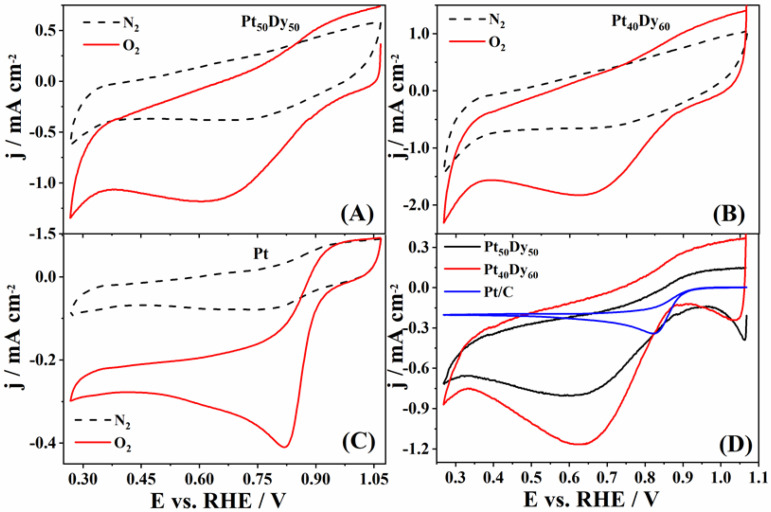
CVs of the Pt_50_Dy_50_ (**A**), Pt_40_Dy_60_ (**B**), and Pt (**C**) electrodes in N_2_- and O_2_-saturated 1 M NaOH and comparison of background current-corrected CVs of three electrodes (**D**) at a scan rate of 10 mV s^−1^ and 25 °C.

A comparison of the voltammograms of Pt–Dy alloys and Pt electrodes in N_2_ and O_2_-saturated solutions showed significant reduction currents attributed to the catalysis of O_2_ reduction, indicating activity toward ORR of the alloy electrodes (Figure 1A–C). The reduction process starts the earliest at the Pt electrode, but both Pt–Dy alloys gave several times higher ORR peak current density than the Pt electrode. Thus, the highest ORR peak current density was obtained for Pt_40_Dy_60_ (−1.16 mA cm^–2^), followed by Pt_50_Dy_50_ (−0.80 mA cm^–2^) and Pt (−0.34 mA cm^–2^) electrodes at 0.64, 0.60, and 0.78 V, respectively, as shown in Figure 1D. Comparing the two Pt–Dy electrodes’ performances for ORR reveals a higher activity of the Pt_40_Dy_60_ electrode, i.e., the electrode with the smaller amount of Pt, Figure 1D. Specifically, the ORR at the Pt_40_Dy_60_ electrode starts at a more positive potential, i.e., lower overpotential than Pt_50_Dy_50_. Furthermore, current densities recorded at the Pt_40_Dy_60_ electrode were higher than those recorded at the Pt_50_Dy_50_ electrode. It is worth noting that a relatively small change in the alloy composition brings a significant difference in the behavior of the alloy towards the ORR. DFT studies were used to shed light on such behavior.

**Figure 2:**
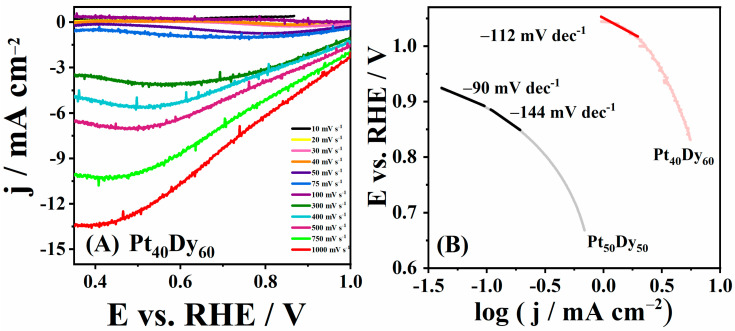
(**A**) Background-corrected LSVs of Pt_40_Dy_60_ alloy electrodes at different polarization rates in O_2_-saturated 1 M NaOH at 25 °C with (**B**) Tafel plots of the two Pt–Dy alloy electrodes.

Subsequently, LSVs of the two Pt–Dy electrodes recorded at different polarization rates revealed that ORR at Pt_40_Dy_60_ starts at ca. 1 V with background-corrected current densities ranging from –0.6 mA cm^–2^ at 0.01 V s^–1^ to –13.5 mA cm^–2^ at 1 V s^–1^, Figure 2A. ORR at Pt_50_Dy_50_ started at ca. 0.86 V with a current density reaching a maximum value of −0.8 mA cm^−2^ at a scan rate of 1 V s^−1^ (not shown). ORR Tafel slopes were determined from the *j*–*E* curves by analyzing the start of the reaction in the kinetic-controlled region, Figure 2B. Tafel slope value for ORR at the Pt_40_Dy_60_ electrode was evaluated to be −0.112 V dec^–1^. In comparison, the value for the Pt_50_Dy_50_ electrode increased from −0.090 to −0.144 V dec^–1^ with the increase in the cathodic overpotential. The evaluated values are similar to those reported for pure Pt [40], thus indicating that the ORR at Pt–Dy alloys most likely proceeds by a mechanism similar to that at Pt. Namely, the ORR Tafel slope at Pt-based electrodes is typically between −0.060 and −0.120 V dec^–1^, i.e., gradually changing between these two values with the increase in cathodic overpotential [40].

The influence of temperature on the ORR at the Pt_40_Dy_60_ electrode was also investigated by recording LSV and CA curves in O_2_-saturated solution in the temperature range 25–65 °C (not shown). As expected, ORR kinetics at the Pt_40_Dy_60_ electrode is enhanced by raising the temperature, as evidenced by increasing current densities. The current density increased from −8 mA cm^–2^ at 25 °C to −37.5 mA cm^–2^ at 65 °C. The activation energy, *E*_a_, of ORR at Pt_40_Dy_60_ was evaluated using the slope of the Arrhenius plot, showing the dependence of j on 1/*T*, and it was found to be 26 kJ mol^–1^. *E*_a_ values in a wide range from 21 to 83 kJ mol^–1^ have been previously reported for ORR at Pt in acidic and alkaline media, depending on the catalyst and method used [41,42,43,44].

**Figure 3:**
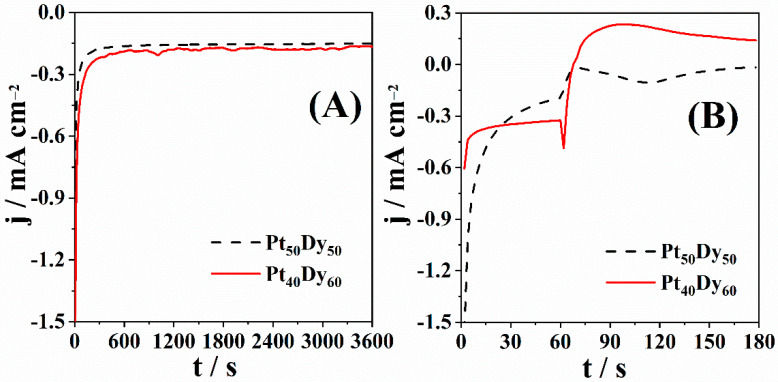
(**A**) Chronoamperometric curves of the Pt–Dy alloy electrodes in O_2_-saturated 1 M NaOH for 1 h at 0.77 V at 25 °C and (**B**) with the addition of fuel solution (0.03 M NaBH_4_) at 0.77 V.

The chronoamperometric measurements revealed short-term stability of Pt–Dy alloys’ activity towards ORR with stable cathodic current densities recorded after the initial drop, Figure 3A. As one of the intended applications of Pt–Dy electrodes was as cathodes in AFCs, and their subclass of direct borohydride fuel cells (DBFCs) with NaBH_4_ as fuel, the selectivity of the Pt_40_Dy_60_ electrode for ORR in the presence of NaBH_4_ was investigated. The chronoamperometric measurements revealed a decrease in cathodic current densities upon the addition of NaBH_4_, Figure 3B. Furthermore, LSV recorded in O_2_-saturated 0.5 M NaBH_4_ + 4 M NaOH solution revealed high positive currents in the expected ORR potential range (not shown). This was somewhat expected as Pt and Pt–Dy alloys were previously reported to be active in the borohydride oxidation reaction [45]. Thus, Pt–Dy electrodes were demonstrated to not be suitable to serve as cathodes in membraneless DBFCs as they are not tolerant to the presence of NaBH_4_.

**Figure 4:**
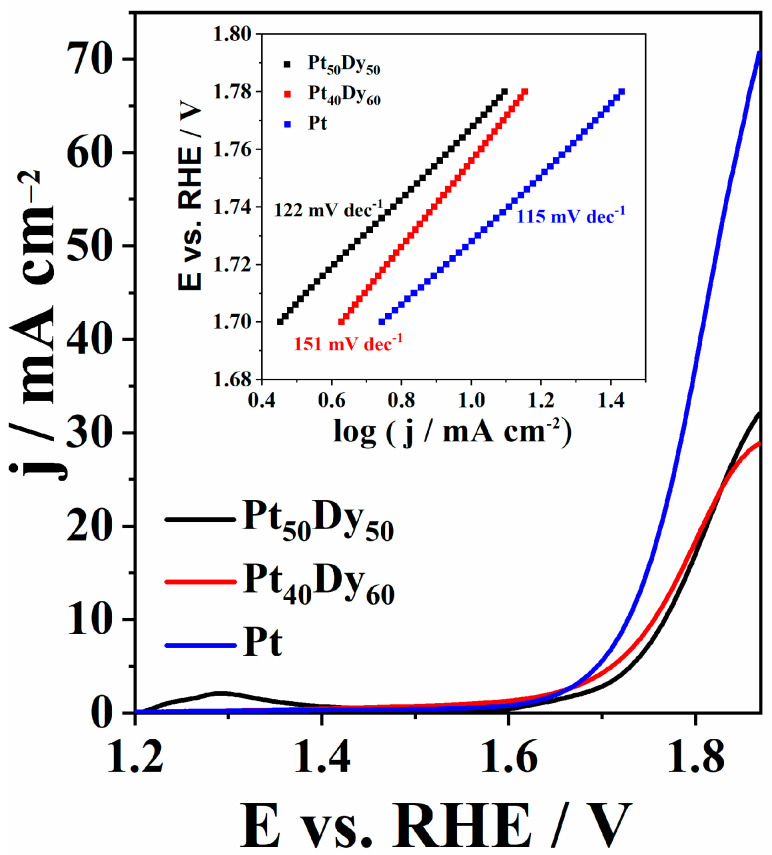
OER polarization curves of the Pt_50_Dy_50_, Pt_40_Dy_60_, and Pt electrodes in 1 M NaOH at a scan rate of 20 mV s^−1^ and 25 °C, with the corresponding Tafel slopes on the inset.

Additionally, the Pt–Dy alloys were examined for OER in alkaline media, and the results were compared with those of a Pt electrode (Figure 4). OER polarization curves of Pt–Dy alloys showed lower OER activity of Pt–Dy alloys than of the Pt electrode, with lower current densities recorded using alloys. This behavior could result from different electronic, chemical, and crystal structures of surface Pt–Dy alloys compared with the pure Pt electrode. Pt–Dy alloys showed a more positive onset potential (E_onset_) toward OER ca. 30 mV than that obtained for Pt (ca. 1.69 V vs. 1.65 V, respectively). Similar Tafel slopes of 0.115 and 0.122 V dec^−1^ were calculated for Pt and Pt_50_Dy_50_ electrodes and a somewhat higher value of 0.151 V dec^−1^ for the Pt_40_Dy_60_ electrode. Furthermore, these values are lower/comparable to that reported for a Pt-modified nickel (Ni) foam electrode, exhibiting a Tafel slope of 144 mV dec^−1^ during OER [27]. 

### 3.2. DFT Analysis of the Compositional Dependence of ORR Activity of Pt–Dy Alloys

**Figure 5:**
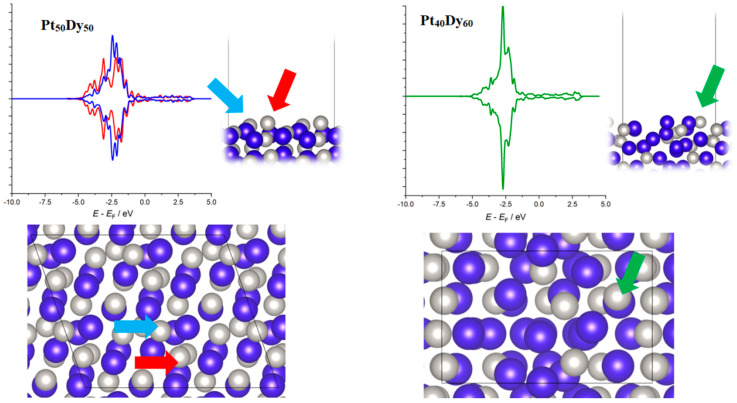
Exposed Pt pair on the Pt_50_Dy_50_ surface (**left**) and exposed Pt atom on the Pt_40_Dy_60_ surface, with the corresponding projected densities of states (PDOS). Grey—Pt, blue—Dy atoms.

To justify the observed compositional sensitivity of ORR on the studied Pt–Dy alloys, we have performed a series of DFT calculations on Pt_50_Dy_50_ and Pt_40_Dy_60_ alloy surfaces. The studied densely packed (002) surfaces expose a small number of Pt atoms, serving as active sites for ORR (Figure 5). While the bands of the majority of Pt atoms in these alloys are greatly stabilized (using the value of the d-band center as a measure of stabilization), on studied Pt_50_Dy_50_ and Pt_40_Dy_60_ alloy surfaces, we have identified pairs and single Pt atoms, respectively, which are exposed to the electrolyte. The pair of Pt atoms in the Pt_50_Dy_50_ alloy surface has d-band centers at −2.12 and −2.25 eV. On the other hand, the exposed Pt atom in the Pt_40_Dy_60_ alloy surface has the band center at −2.40 eV (Figure 5).

The distance between Pt atoms in the Pt–Pt pair on the Pt_50_Dy_50_ surface is 2.8 Å, which is close to that in bulk Pt. However, the exposed Pt atom on the Pt_40_Dy_60_ surface has the nearest Pt neighbor at 3.8 Å, suggesting that Pt atoms in Pt_40_Dy_60_ can be considered single atoms dispersed into the Dy phase. Indeed, the PDOS of the exposed surface Pt atom in the Pt_40_Dy_60_ surface is very sharp and resembles that of single-atom catalysts. This feature is unique and suggests that the usual connections between electronic structure and reactivity could be broken for Pt–Dy alloys.

**Figure 6:**
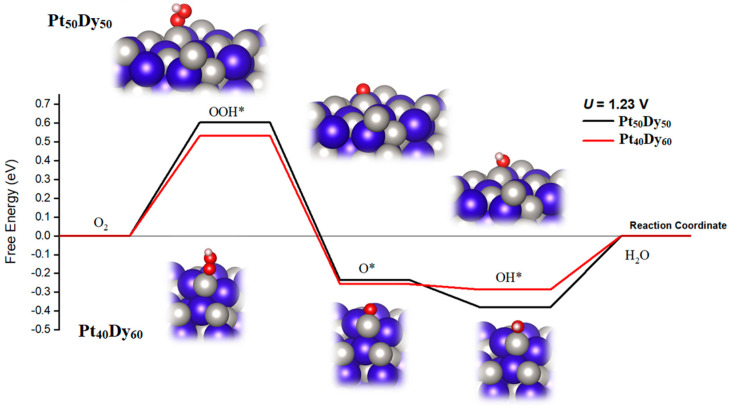
ORR (direct reaction) or OER (reverse reaction) free energy profiles for Pt_50_Dy_50_ and Pt_40_Dy_60_ phases at equilibrium potential, along with the optimized structures of ORR intermediates (OOH, O, and OH) on studied surfaces. Grey—Pt, blue—Dy atoms, * refers to adsorbed species.

We have further investigated the interactions of ORR intermediates with the identified Pt centers (Pt-pair and exposed single Pt atom) to assess the ORR activity of Pt_50_Dy_50_ and Pt_40_Dy_60_ phases (Figure 6).

## 4. Discussion

**Scheme 1:**
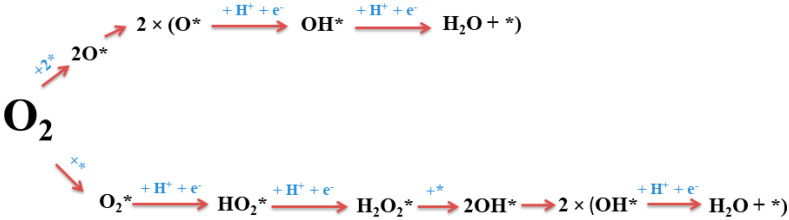
Possible oxygen reduction mechanisms, * refers to adsorbed species.

Two ORR mechanisms on Pt and Pt alloy electrodes were proposed based on both experimental and theoretical studies [9]: direct four-electron mechanism (O_2_ is reduced to H_2_O) and a series two-electron pathway (O_2_ is reduced to H_2_O via H_2_O_2_), Scheme 1. O_2_ can be reduced to H_2_O either through associative or dissociative pathways, with the generation of *OOH (* refers to adsorbed species) from the addition of hydrogen to O_2_ or the generation of 2*O by O_2_ dissociation, respectively [46]. This direct four-electron mechanism is believed to be dominant both in acidic and alkaline media when there are minimal adsorbed impurities [47]. Still, the series ORR pathway may also proceed at the Pt- and Pt alloy electrodes [48], as well as a parallel pathway in which both the direct and series pathways take place simultaneously [49]. The ORR pathway at Pt is also reported to be influenced by the adsorption and coverage of oxygen on its surface so that the direct pathway is dominant at low oxygen coverage (*θ*_O_ = 0 monolayer) and the series pathway is dominant at high oxygen coverage (*θ*_O_ = 0.5 monolayer) [49]. A Tafel slope of −0.060 V dec^–1^ indicates that the rate-determining step (RDS) is a pseudo 2-electron reaction, while a value of −0.120 V dec^–1^ demonstrates that the first electron transfer is the RDS.

The analysis of free energy profiles suggests that the first electron transfer is a potential determining step, in agreement with the experimental results. Moreover, the Pt_40_Dy_60_ phase has a lower thermodynamic barrier for ORR by 0.07 eV, suggesting that ORR should be faster on Pt_40_Dy_60_ catalysts. Based on the reduction in the barrier, catalytic activity enhancement of the Pt_40_Dy_60_ phase compared to Pt_50_Dy_50_ should be increased by a factor of 16. Considering that the density of Pt pairs on the Pt_50_Dy_50_ surface is two times higher than that of Pt single atom sites on the Pt_40_Dy_60_ surface, DFT calculations suggest an eight times higher ORR activity of the Pt_40_Dy_60_ catalyst compared to that of Pt_50_Dy_50_, which agrees well with the experimental results. Note that we considered an associative mechanism of ORR, and, compared to Pt(111), the reactivity is significantly altered by alloying with Dy [37]. Specifically, the first step on Pt(111), the formation of adsorbed OOH, is exergonic compared to the studied Pt–Dy surfaces [37]. However, we also noted that the unique coordination of single Pt atoms on the Pt_40_Dy_60_ surface renders a characteristic behavior. Compared to the Pt_50_Dy_50_ phase, OOH is bonded stronger, but OH is bonded weaker, which also points to different oxophilicities of the Pt_40_Dy_60_ surfaces and lower surface blockage by OH_ads_ in the case of Pt_40_Dy_60_. Considering the OER, the reaction profile (Figure 6) suggests a rather high OER overpotential, amounting to 0.84 and 0.79 V for Pt_50_Dy_50_ and Pt_40_Dy_60_, respectively. This indicates a lower OER activity compared to Pt [37] and somewhat higher OER activity of Pt_40_Dy_60_ compared to Pt_50_Dy_50_, which is in agreement with the experimental findings (Figure 4). In the case of OER, weak OOH bonding is the main limiting factor. Namely, if one considers Figure 6 in the OER direction (from H_2_O to O_2_), it is clear that a large exergonic step going from O* to OOH* determines the OER overpotential. In the case of the Pt_40_Dy_60_ catalyst, this step gives a lower barrier, and thus, OER activity is expected to be somewhat higher. In general, the ORR/OER reaction profile for the Pt_40_Dy_60_ catalyst is smoother compared to the Pt_50_Dy_50_ one, making it overall better for O_2_ reduction/H_2_O oxidation. Further improvements in catalytic activity would require additional flattening of the reaction profile. Namely, to boost both ORR and OER activity, one would require to (i) additionally stabilize *OOH and (ii) destabilize both *O and *OH. The first point seems to be of more crucial importance as it largely relates to the improvement of OER activity, while ORR activity of the studied Pt–Dy catalysts is already rather high (Figure 1).

We believe that finding the single-atom-like behavior of Pt in Pt_40_Dy_60_ is very important as the conventional Pt-based catalysts often suffer from low Pt utilization efficiency as only part of the Pt atoms on the surface are taking part in the catalysis [50]. Therefore, catalysis by single Pt atoms is probably recognized as the most efficient approach to bring the Pt utilization efficiency close to 100% and, thus, reduce the cost of Pt catalysts. The single-atom catalysts have exceptional mass activity by exploiting low-coordination and unsaturated active sites. The *d* orbitals in single-atom catalysts have been reported to be easily split and tuned (conversely to bulk systems), resulting in high performance for the ORR of a single-atom, paired catalyst and equivalent systems [46]. This could lead to a strategy to further reduce cathode catalyst costs, such as making core-shell systems with a Pt_40_Dy_60_ core, where the core price would be reduced by alloying with Dy. At the same time, the single-atom-like behavior of the surface Pt atoms would ensure a high ORR activity. 

## 5. Conclusions

The electrochemical activity of Pt–Dy alloy electrodes of two different compositions towards the ORR was examined. Voltammetry and chronoamperometry analysis revealed that Pt–Dy electrodes are active for the ORR, with the Pt_40_Dy_60_ electrode exhibiting substantially higher activity than Pt_50_Dy_50_. The Tafel slope values for Pt–Dy alloys were comparable to those reported for pure Pt, indicating that ORR at these alloys follows the same pathway as at Pt. DFT calculations revealed the origin of higher ORR activity of the Pt_40_Dy_60_ electrode. A unique single-atom alloy-like coordination and electronic structure of surface Pt atoms render stronger OOH and weaker OH bonding to the Pt_40_Dy_60_ surface, compared to the bonding towards Pt_50_Dy_50_, in both cases roughly by 0.1 eV, making the former approximately eight times more active. Pt–Dy alloys also showed a reasonable OER activity, though lower than the Pt electrode. It is worth noting that the electrode with a lower Pt content, and thus lower price, showed higher activity for ORR, offering a potential solution for lowering the fuel cells’ cathode price (as Dy is 85 times cheaper than Pt) while maintaining a high ORR activity.

## Data Availability

Data available on request of the corresponding author.
